# Clinical Remarks. Professor Thomas F. Rochester, on Pulmonary Tuberculosis

**Published:** 1871-03

**Authors:** F. Bradnack

**Affiliations:** Late Member of the Class


ART. IV.—*Clinical Remarks.* Professor THOMAS F. ROCHESTER on Pulmonary Tuberculosis.

REPORTED BY F. BRADNACK, M. D., (LATE MEMBER OF THE CLASS.)

The patient we have just seen has tubercles in both lungs. This disease (tuberculosis or phthisis) is of all others, the one which preeminently characterizes civilization. Savage races are never, or rarely, afflicted by it; and this remark likewise applies to semi-barbarous races, with the exception of the instances in which the white man has gone, and carried his vices among these people. For instance: the crew of Captain Cook carried syphilis to the Sandwich Islands; the result being that these Islanders now possess, to a marked degree, the syphilitic cachexia, and following, as a natural sequence, comes tuberculosis, which fastens itself freely upon these patients; it being now well-known that tuberculosis is very general in these islands. And this series of evils is found to result in like manner everywhere, under the same circumstances. The Chinese suffer from terrible forms of syphilis; and pulmonary tuberculosis likewise exists there. That the existence of syphilis predisposes to tuberculosis, cannot be doubted. Sufficient attention has not as yet been paid to this fact. Phthisis is also, in all probability, engendered in luxury, especially the more enervating forms of the latter: e. g., late hours, insufficient bodily exercise, indolence. &c. It may likewise probably, be caused by excessive mental labor, overworking the brain at the expense of the rest of the body; also, by bad ventilation, (the evils of which latter can scarcely be stated in too strong terms, or pointed out too frequently.) Phthisis annually carries off large numbers of the most cultivated people of the globe. This is a patent and undeniable fact. It appears that any cause, or series of causes, which depresses or enervates, or which tends to depress or enervate the physical system, favors the production of tubercle. Without attempting a full explanation, this may be accepted as a fact. For instance: during the famine fever in Ireland, the mental depression and discontent, together with the physical evils, greatly favored the progress of, and the mortality from phthisis.

I have no doubt that the disease is hereditary, yet many think otherwise; though it seems impossible to see how they can think so. I have in my cabinet the lungs of an infant a few weeks old, which contain a cavity formed during intra-uterine life. It, doubtless, may sometimes happen that, where one parent only has tuberculosis, the disease *may* not be developed in the offspring; but, if both parents are tuberculous, the children will almost surely be so. The tuberculous diathesis is supposed to be indicated by long, taper fingers, blue eyes, and pearl-colored sclerotic and fair skin. This, therefore, must be regarded as a fatal kind of beauty.

Pulmonary tuberculosis is a variable affection. After a period of activity comes a period of rest. It is asked: what *is* tubercle? The tubercle is an exudate from the blood, thrown out in a liquid form, the firmer portions of which remain as granules. While the blood is passed through the lungs to be oxygenated, the deposit takes place. It occurs in the air tubes, and in their lining membrane, and also outside the air tubes. Tubercle has no power of self-increase, but its presence furnishes a point of irritation. The deposit is said to occur more frequently in the left lung; but I believe the left lung is not so primarily affected as is imagined. Phthisis is sometimes styled an inflammatory affection. Professor Niemeyer so denominates it. This author asseverates that tubercle is often nothing more than pus and lymph. But I am compelled to dissent from this conclusion. The fact that we find tubercles in every portion of the body, would appear to be a sufficient refutation of the proposition, inasmuch as this fact strongly indicates the deposit to be an exudation. Some authors have thought that because phthisis is occasionally ushered in by hœmoptysis, that the latter is the cause of the disease, but this reasoning is manifestly illogical.

